# Depressive and PTSD symptoms are associated with regional differences in light-evoked brain responses in chronic ocular pain

**DOI:** 10.21203/rs.3.rs-11053966/v1

**Published:** 2026-10-11

**Authors:** Eric Moulton, Ema Karakoleva, David Valdes-Arias, Pradip Pattany, Vanda Faria, Elizabeth Helix, Anat Galor

**Affiliations:** Boston Children’s Hospital and Harvard Medical School; Miami Veterans Affairs Medical Center; Miami VAMC

## Abstract

**Background/Objectives:**

Chronic ocular pain (COP) often co-occurs with depression or post-traumatic stress disorder (PTSD), but whether these comorbidities confer a distinct biological signature is unknown. Thus, we compared light-evoked blood oxygen level-dependent (BOLD) responses in individuals with COP, stratified by the presence or absence of clinically relevant depressive and/or PTSD symptoms.

**Subjects/Methods:**

43 individuals with COP (pain intensity ≥ 1/10 for ≥ 3 months) underwent event-related functional magnetic resonance imaging using bright-light. Thirty participants were classified as COP with mental health comorbidity (COP + MHC+) based on a Patient Health Questionnaire-9 (PHQ-9) score ≥ 5 and/or a PTSD Checklist-Military Version (PCL-M) ≥ 33; 13 were classified as COP without MHC (COP + MHC-). BOLD responses exceeding an exploratory voxelwise threshold of Z > 2.3 were compared within predefined bilateral regions of interest (ROIs) comprising the anterior cingulate cortex (ACC), anterior mid-cingulate cortex (aMCC), anterior insula, and amygdala. Regional parameter estimates were correlated with clinical symptoms.

**Result:**

Demographics were similar between groups. Participants with COP + MHC+ reported greater dry-eye symptom burden and non-ocular pain, whereas measured ocular surface signs and in-scanner eye-pain and unpleasantness ratings did not differ significantly between groups. In exploratory ROI analyses, COP + MHC+ participants demonstrated greater light-evoked BOLD responses in the right ACC and caudal right aMCC, whereas COP + MHC− participants demonstrated greater responses in the rostral right aMCC and right anterior insula.

**Conclusions:**

These findings suggest that depressive and PTSD symptoms may contribute to heterogeneity in central pain processing among individuals with COP. If confirmed, such differences could help refine COP phenotyping and inform more targeted, mechanism-based treatment strategies.

## Introduction

Chronic ocular pain (COP) is a condition that may variably involve nociceptive, neuropathic, and/or nociplastic mechanisms and can substantially impair quality of life.(1–3) It is characterized by spontaneous or evoked dysesthesias, that can include dryness, aching, and burning, with wind and light as common triggers.(4, 5) COP rarely occurs in isolation and frequently coexists with other chronic pain syndromes (CPS) and mental health comorbidities (MHC).(6–9) Population-based studies in Veterans Affairs cohorts demonstrated higher rates of depression and post-traumatic stress disorder (PTSD) among individuals coded for dry eye disease (DED)(7), a diagnosis that may serve as a surrogate for COP in administrative datasets. This relationship persisted in clinical cohorts. In 154 South Florida Veterans with symptoms of ocular discomfort and dryness, individuals with a high versus low burden of CPS reported more severe neuropathic ocular pain symptoms, measured using the Neuropathic Pain Symptom Inventory-Modified for the Eye (NPSI-Eye), despite similar ocular surface findings, and also endorsed higher levels of depression and PTSD symptoms.(9) The association between ocular pain, particularly light sensitivity, and mental health comorbidities also extends to individuals with migraine(10) and fibromyalgia (FM).(11) Collectively, these observations suggest that light sensitivity and greater patient-reported symptom burden frequently co-occur with psychiatric symptoms across several chronic pain populations.

Functional magnetic resonance imaging (fMRI) provides a means of examining how light engages neural systems involved in sensory and pain processing. In migraine, photophobia has been linked to interactions between visual and trigeminal nociceptive pathways(8), and visual-stimulation fMRI during migraine attacks has demonstrated responses within cortical pain-related regions including the anterior cingulate cortex (ACC), mid-cingulate cortex, and insula.(12) In FM, neuroimaging studies have reported differences in affective pain-processing networks between individuals with FM and healthy controls, although findings vary across task-based and resting-state paradigms.(13, 14) Meta-analytic resting-state work across chronic pain conditions has likewise implicated regions involved in affective pain processing.(15) Collectively, these findings demonstrate overlap between brain regions involved in pain processing and those engaged during light-evoked responses. Because depression and PTSD symptoms commonly co-occur with chronic pain and engage many of the same brain regions(7, 16–18), there is biological plausibility that these symptoms may contribute to variability in light-evoked neural responses.

In prior work using a light-evoked fMRI paradigm, individuals with COP and photoallodynia, defined as pain elicited by normally non-painful light, showed enhanced activation within trigeminal brainstem nuclei, visual and somatosensory cortices, the anterior mid-cingulate cortex (aMCC), and anterior insula compared with pain-free controls.(19) Several of these regions are also involved in affective-motivational and salience-processing across CPS, migraine, and mood- and trauma-related disorders.(15, 18, 20, 21) However, whether depressive or PTSD symptoms are associated with variability in these light-evoked responses in COP remains unknown. Because depressive and PTSD symptoms substantially overlapped in the present cohort, we examined them primarily as a combined screen-positive phenotype rather than attempting disorder-specific comparisons, while also examining PHQ-9 and PCL-M scores dimensionally. We therefore used an *a priori* region-of-interest approach focused on the bilateral ACC, aMCC, anterior insula, and amygdala. We hypothesized that participants with COP who screened positive for depressive and/or PTSD symptoms (COP + MHC+) would exhibit greater light-evoked BOLD responses within these regions than participants who screened below both thresholds (COP + MHC−).

## Methods

### Study Registration, Ethical Approval, and Consent Procedures.

The study was approved by the Institutional Review Boards (IRBs) of the Miami Veterans Affairs (VA) Medical Center and the University of Miami (IRB approvals #3011.08 and 20190340, respectively). The study adhered to the ethical guidelines in the Declaration of Helsinki and complied with the United States Health Insurance Portability and Accountability Act requirements. Prior to the initiation of any study activities, written informed consent was obtained from all participants.

### Study Participants.

Participants were recruited from the Miami VA eye clinic. Baseline data from sixteen participants were previously included in a separate study evaluating the effects of botulinum toxin A on pain- and photophobia-related neural pathways in COP using fMRI.(22) That study examined treatment-related longitudinal changes, whereas the present analysis focuses exclusively on how depressive and PTSD symptoms modulate light-evoked brain responses at baseline. Although baseline data from 16 participants were included in the prior treatment study, the present analysis addresses a distinct question, uses a different grouping variable, and reports no previously published between-group comparisons.

Inclusion criteria required the presence of COP, defined as an average ocular pain intensity ≥ 1 on a 0–10 numeric rating scale (NRS) over the past week and a pain duration of ≥ 3 months. Exclusion criteria targeted ocular or systemic factors that could confound the assessment of COP or photoallodynia, including glaucoma (or its pharmacological treatment), uveitis, iris transillumination defects, retinal degeneration, or structural abnormalities of the cornea, conjunctiva, or eyelids. Individuals with standard contraindications to MRI, such as pacemakers, implanted metal hardware, or pregnancy, were also excluded. Notably, medication use and systemic comorbidities were not exclusion criteria.

Participants were stratified based on the presence or absence of mental health comorbidity (MHC). Depression symptoms were assessed using the Patient Health Questionnaire-9 (PHQ-9; range 0–27), with higher scores indicating greater symptom severity. Depressive symptoms were categorized as screen-positive at PHQ-9 ≥ 5, corresponding to at least mild symptom severity.(23) PTSD symptoms were assessed using the PTSD Checklist-Military Version (PCL-M; range 17–85), with higher scores indicating greater symptom severity. PTSD symptoms were categorized as screen-positive at PCL-M ≥ 33.(24)

### Participant Groups.

For analytic purposes, participants screening positive for depressive symptoms (PHQ-9 ≥ 5) and/or PTSD symptoms (PCL-M ≥ 33) were categorized as COP + MHC+. Participants scoring below both thresholds were categorized as COP + MHC−. The term MHC is used as analytic shorthand and does not indicate a psychiatric diagnosis. Participants were stratified into those with COP who screened positive for depressive and/or PTSD symptoms (COP + MHC+, n = 30) and those who scored below both screening thresholds (COP + MHC−, n = 13). This stratification allowed us to examine associations between psychiatric symptom burden and affective-motivational components of pain processing. Depression and PTSD symptoms were combined because of their substantial overlap in the cohort: 27 of 30 COP + MHC+ participants met both thresholds. The combined grouping therefore reflects a predominantly co-occurring depressive/PTSD symptom phenotype and does not permit disorder-specific inference. To preserve dimensional information, PHQ-9 and PCL-M scores were also examined as continuous symptom measures in correlation analyses.

### Study design.

During a dedicated clinical visit, participants completed detailed questionnaires assessing demographics, medical history, systemic and ocular medication use, and ocular pain characteristics. Each visit included a standardized ocular surface examination and additional validated surveys evaluating ocular pain, light sensitivity, and dry eye symptoms.

### Questionnaires and Psychometric Scales.

Ocular pain and related symptoms were assessed using multiple validated tools. The Numerical Rating Scale (NRS; range 0–10) measured ocular pain intensity. Neuropathic ocular pain symptoms were assessed using select items from the NPSI-Eye (range 0–100), specifically items evaluating pain provoked or worsened by wind (question [Q] 8), light (Q9), and temperature changes (Q10).(25, 26) Photoallodynia severity was assessed using the Visual Light Sensitivity Questionnaire-8 (VLSQ-8; range 8–40).(27) DED symptoms were evaluated using the Ocular Surface Disease Index (OSDI; range 0–100), which assesses symptom severity, environmental triggers, and impact on visual functioning(28), and the 5 Item Dry Eye Questionnaire (DEQ-5; range 0 − 22), which focuses on dryness, discomfort, and tearing independent of vision.(29) Depressive and PTSD symptoms were assessed using the PHQ-9 and PCL-M, respectively, as described above.

### Ocular Surface Examination.

A standardized ocular surface examination was performed to characterize and compare ocular surface signs between MHC groups. Slit-lamp biomicroscopy was performed to document eyelid and conjunctival findings. Ocular surface signs were graded using established scales (higher scores indicating greater severity) for anterior blepharitis (0–3), eyelid vascularity (0–3), palpebral hyperemia (0–2), conjunctival papillae (0–2), meibomian gland plugging (0–3), and meibum quality (0–4). Tear film stability was assessed by instilling one drop of preservative-free fluorescein and measuring tear breakup time (TBUT) as the average of three readings per eye (in seconds). Corneal staining was graded in five corneal regions on a 0–3 scale (total 0–15) using the National Eye Institute (NEI) scoring system to quantify epithelial irregularity.(30) Ocular pain intensity (0–10 NRS scale) was recorded before and after instillation of one drop of 0.5% proparacaine in each eye. Scores were analyzed at baseline, post-anesthetic application, and as the difference (post-anesthetic minus baseline). Persistent pain after topical anesthesia has been proposed to reflect a non-ocular or more centrally mediated pain source.(31) Finally, basal tear production was assessed using Schirmer’s strips placed in the lower conjunctival fornix for five minutes, with wetting length recorded in millimeters (mm); values < 5 mm were considered indicative of low tear production.

### MRI Acquisition.

The fMRI protocol for COP and photophobia was adapted from prior work.(32) All participants underwent MRI at the University of Miami Coral Gables campus using a 3T Siemens MAGNETOM Vida scanner (Erlangen, Germany) equipped with a Siemens BioMatrix Head/Neck 20-channel coil. High-resolution anatomical images were acquired using a sagittal three-dimensional T1-weighted Magnetization Prepared Rapid Gradient Echo (MPRAGE) sequence (TE/TR = 2.38/2100ms; 192 slices; 1.00-mm slice thickness; 1.00 × 1.00 mm in-plane resolution; matrix, 256 × 256. Functional images were collected using a gradient-echo (GE) echo-planar imaging (EPI) sequence (TR/TE = 2100/30 ms; 290 volumes; 100 oblique slices; 1.50-mm slice thickness; 1.94 × 1.94 mm in-plane resolution). Slices were aligned to the long axis of the caudal brainstem to optimize signal acquisition in inferior brain regions.(32)

During fMRI, participants viewed intermittent bright light in a darkened environment under two conditions: OFF (black screen with white fixation cross, ~ 0.5 lux) and ON (white screen with black fixation cross, ~ 65 lux). Bright-light ON blocks (6 s) were separated by OFF intervals of 26–34 s, jittered in 2-s increments to minimize anticipatory effects. One drop of Refresh Plus Lubricant Eye Drops (Allergan) was administered in both eyes before each scan. Participants kept their eyes open and blinked normally throughout. Light-evoked blood oxygen level dependent (BOLD) responses were analyzed as an indirect measure of task-related neural activity. At the end of each scan, participants rated light-evoked eye pain intensity and unpleasantness on separate 0–100 numeric rating scales.

### Image Analysis: Preprocessing.

Functional and anatomical images were converted from Digital Imaging and Communications in Medicine (DICOM) to Neuroimaging Informatics Technology Initiative (NIfTI) format prior to analysis. Functional imaging preprocessing was then performed using the Functional MRI of the Brain (FMRIB) Software Library (FSL v6.0.7.15; Oxford Centre for Functional MRI of the Brain, University of Oxford). Brain extraction was performed on each subject’s T1-weighted MPRAGE image using FSL’s Brain Extraction Tool (BET). The first three EPI volumes were discarded to allow for signal equilibration, and functional data were motion-corrected using FMRIB’s Linear Image Registration Tool (MCFLIRT). Scans exceeding motion thresholds (> 1.5 mm relative translation, > 1° rotation between volumes, or > 3 mm absolute translation across the scan) were excluded. Subsequent preprocessing included spatial smoothing (5-mm Gaussian kernel), high-pass temporal filtering (sigma = 50 s), and normalization to the Montreal Neurological Institute (MNI152) standard template (2-mm isotropic resolution) for group-level analyses.

### ROI-based Analyses.

Analysis was restricted to *a priori* regions of interest (ROIs) selected for their relevance to affective-motivational pain processing: bilateral anterior cingulate cortex (ACC), anterior mid-cingulate cortex (aMCC), anterior insula, and amygdala. These regions were selected because the ACC contributes to affective pain processing and shows altered activation in mood- and trauma-related disorders,(18, 21, 33, 34) the aMCC supports pain-related cognitive control and motivation,(34) the anterior insula integrates interoceptive and salience-related signals,(35–37) and the amygdala mediates threat detection and emotional salience and demonstrates heightened reactivity in trauma-related conditions.(21, 38, 39) ROIs were defined in MNI152 standard space using the Harvard-Oxford cortical and subcortical atlases distributed with FSL, with probability maps thresholded at ≥ 20% to balance anatomical specificity with spatial coverage. Hemisphere-specific masks were generated by dividing each atlas-derived ROI mask at the midsagittal plane, and cluster locations were anatomically verified by visual inspection using structural overlays.

Within these ROIs, a General Linear Model (GLM) was implemented using FEAT (FMRI Expert Analysis Tool) with FLAME (FMRIB’s Local Analysis of Mixed Effects) to examine group differences in light-evoked BOLD responses. Two directional contrasts were evaluated: COP + MHC + > COP + MHC− and COP + MHC−> COP + MHC+. Voxelwise results were examined at Z > 2.3 with a minimum cluster extent of six contiguous voxels. For each contrast, analysis was restricted to voxels showing positive light-evoked activation exceeding the specified voxelwise threshold in the reference group (COP + MHC+ for the first contrast and COP + MHC− for the second). No correction for multiple comparisons across voxels or ROIs was applied; therefore, these ROI analyses were considered exploratory and hypothesis-generating. For each retained cluster, contrast of parameter estimate (COPE) values, representing the magnitude and direction of BOLD signal differences, were averaged for visualization.(40) Subject-level parameter estimate (PE) values were extracted from first-level COPE images within each retained cluster and used for correlations with clinical measures.

### Whole-brain Analysis.

An exploratory whole-brain analysis was performed using a cluster-forming voxelwise threshold of Z > 2.3 with cluster-level family-wise error correction at p < 0.05. Cluster locations were identified using the Harvard-Oxford cortical and subcortical atlases in FSL.

### Statistical Analysis

Statistical analyses were performed using SPSS v29.0 (Armonk, NY, USA). Descriptive statistics summarize demographic, clinical, and ocular surface characteristics. Between-group comparisons (COP + MHC− vs. COP + MHC+) were conducted using independent-samples *t*-tests, Mann-Whitney U tests, or Fisher’s exact tests, as appropriate. Nonparametric tests were used when normality assumptions were violated. For clusters exceeding the exploratory threshold, extracted PE values were correlated with PCL-M, PHQ-9, and NPSI-Eye Q9 scores using Spearman’s rank-order correlations (*ρ*). These correlations were considered descriptive post hoc analyses. To account for multiple testing, p-values for these correlations were adjusted using the Benjamini-Hochberg false discovery rate (FDR) procedure, with FDR-adjusted *q* < 0.05 considered statistically significant. Because the clusters were identified on the basis of MHC group contrasts, correlations with the group-defining PHQ-9 and PCL-M measures were not statistically independent of cluster selection and were therefore interpreted descriptively rather than as independent confirmation of the group findings.

## Results

Of the 61 individuals with chronic ocular pain (COP) who completed clinical examination and fMRI scanning, 43 (mean age: 55.9 ± 11.5 years; 37% female) were included in the final analysis. Eighteen participants were excluded because of scan corruption (n = 4), excessive motion artifact (n = 10), absence of a detectable occipital visual-cortex response during light stimulation (n = 3), or incidental intracranial findings (n = 1).

Participants were categorized as COP + MHC+ (n = 30; mean age: 56.7 ± 11.5 years; 30% female) if they screened positive for depressive and/or PTSD symptoms and as COP + MHC− (n = 13; mean age: 54.2 ± 11.9 years; 54% female) if they screened below both thresholds. Age, sex, race, ethnicity, and most systemic comorbidities did not differ significantly between groups (Table 1).

### Symptom and Ocular Surface Characteristics.

Compared with COP + MHC− participants, those with COP + MHC+ generally reported greater ocular symptom severity, most notably higher OSDI scores (60.6 ± 19.5 vs. 41.0 ± 17.4, p = 0.003), whereas other ocular symptom measures did not differ significantly between groups (Table 2). Ocular pain ratings obtained during the examination were also higher in the COP + MHC+ group both before (3.8 ± 2.8 vs. 1.8 ± 2.7, p = 0.03) and after (2.7 ± 2.7 vs. 0.8 ± 1.0, p = 0.02) topical anesthesia, although the proportion of participants reporting any residual pain after anesthesia did not differ between groups. Participants with COP + MHC+ also reported greater non-ocular pain intensity during the preceding week, including higher worst pain (6.8 ± 2.6 vs. 3.9 ± 3.8, p = 0.02) and average pain (5.6 ± 2.5 vs. 3.4 ± 3.3, p = 0.047). In contrast, tear parameters and other measured ocular surface signs did not differ significantly between groups (Table 2).

### ROI-Based fMRI Findings.

Exploratory ROI analysis identified right-sided clusters exceeding the voxelwise threshold within the ACC, aMCC, and anterior insula (Fig. 1A; Table 3). Within the right aMCC ROI, two spatially distinct clusters emerged in opposite group contrasts. COP + MHC+ participants demonstrated greater light-evoked BOLD responses in the right ACC and the more caudally located right aMCC cluster, whereas COP + MHC− participants demonstrated greater responses in the more rostrally located right aMCC cluster and right anterior insula. Because the aMCC subregions were not prespecified and the Harvard-Oxford atlas does not provide separate rostral and caudal aMCC labels, these clusters are referred to descriptively throughout the manuscript as the caudal and rostral right aMCC, respectively. No clusters exceeding the exploratory threshold were identified within the amygdala. Peak MNI coordinates, Z statistics, and cluster extents are summarized in Table 3.

In descriptive post hoc analyses, extracted cluster parameter estimates were correlated with selected clinical measures (Fig. 1B). ACC BOLD responses were positively correlated with PCL-M (*ρ* = 0.43, p = 0.004) and NPSI-Eye Q9 scores (evoked pain to light; *ρ* = 0.38, p = 0.01). Caudal aMCC responses were positively correlated with PHQ-9 scores (*ρ* = 0.34, p = 0.02) only, whereas rostral aMCC responses were inversely correlated with both PCL-M (*ρ*=−0.47, p = 0.001) and PHQ-9 scores (*ρ*=−0.44, p = 0.003). Anterior insula responses were inversely correlated with NPSI-Eye Q9 (*ρ*=−0.33, p = 0.03), PCL-M (*ρ*=−0.64, p < 0.001), and PHQ-9 scores (*ρ*=−0.44, p = 0.003). All nominally significant correlations remained significant after FDR correction (*q* < 0.05).

### Exploratory Whole-Brain Analysis.

Whole-brain analysis identified two clusters surviving cluster-level correction for the COP + MHC− > COP + MHC+ contrast, including a large bilateral occipital-posterior cortical cluster involving the supracalcarine cortex, cuneus, lingual gyrus, fusiform gyrus, precuneus, intracalcarine cortex, and posterior cingulate gyrus, as well as a left occipital-angular gyrus cluster. No clusters survived correction for the opposite contrast. Detailed cluster coordinates and statistics are reported in **Supplementary Table S1**.

## Discussion

When examining the clinical and neurobiological characteristics of individuals with COP with and without MHC, differences emerged across both domains. Clinically, the COP + MHC+ group reported greater ocular symptom burden on select measures and greater non-ocular pain intensity despite similar ocular surface signs. Interestingly, in-scanner ratings of eye pain and unpleasantness did not differ significantly between groups. Neurobiologically, however, COP + MHC+ participants demonstrated a distinct regional pattern of light-evoked BOLD responses compared with those who screened below both mental health thresholds. These findings partially supported our directional hypothesis: COP + MHC+ participants showed greater responses in the right ACC and caudal right aMCC, whereas differences in the rostral right aMCC and right anterior insula were in the opposite direction. In descriptive post hoc analyses, regional responses were correlated with psychiatric symptom severity and light-related pain in directions generally consistent with the group contrasts. Together, these findings suggest that psychiatric symptom burden may represent an important source of heterogeneity in light-evoked neural responses among individuals with COP.

The greater symptom burden observed in COP + MHC+ is consistent with findings in other chronic pain disorders, including migraine and fibromyalgia (FM). In migraine, depression and anxiety are common comorbidities that have been associated with greater pain burden(16); for example, among individuals with migraine and interictal photophobia, greater depressive and anxiety symptom severity has also been associated with greater photophobia severity, suggesting a relationship between psychiatric symptom burden and light sensitivity.(10) Similarly, depression and anxiety are highly prevalent among individuals with FM,(41) and greater depressive symptom burden has also been associated with increased pain intensity, functional impairment, and poorer quality of life in FM.(17, 42) Consistent with these observations, participants with MHC in our cohort reported greater ocular symptom burden on some, but not all, measures. Interestingly, light sensitivity assessed using multiple questionnaire measures did not differ significantly between groups, an unexpected finding that underscores the clinical complexity and multidimensional nature of pain.

From a neuroimaging perspective, the present findings can be interpreted in the context of prior neuroimaging studies in other chronic pain disorders. In migraine, visual-stimulation fMRI during attacks has demonstrated responses within cortical pain-related regions including the ACC, mid-cingulate cortex, and insula(12), while photophobia has been linked to interactions between visual and trigeminal nociceptive pathways.(8) Although COP differs clinically from these disorders, the overlap in light-responsive regions suggests that light stimulation may engage partially shared pain-processing networks across chronic pain conditions. Importantly, however, measures of light sensitivity did not differ significantly between the COP + MHC+ and COP + MHC− groups in the present study. Thus, the observed neural differences were not accompanied by greater self-reported photoallodynia in the MHC group, suggesting that psychiatric symptom burden may be associated with differences in neural responses to light even in the absence of differences in reported light sensitivity. Psychiatric symptom burden has likewise been associated with altered activity within affective pain-related circuits in FM. Pressure-pain fMRI studies demonstrated that greater depressive symptom burden was associated with increased pain-evoked activation of the amygdala and contralateral anterior insula, whereas primary and secondary somatosensory cortices were not associated with depression status.(42, 43) Complementary resting-state and emotion-processing studies have also reported altered ACC connectivity associated with depression and anxiety severity. (13) Although these studies used pressure-pain, resting-state, or emotion-processing paradigms rather than photic stimulation, they collectively suggest that psychiatric symptoms are associated with selective alterations in affective pain-processing circuits rather than generalized changes across all pain-related regions. Within COP, prior light-evoked fMRI work identified greater aMCC and anterior insula responses relative to pain-free controls(19), and the present study suggests that depressive and/or PTSD symptom burden may be associated with region-specific variability within these same pain-processing regions. Taken together, COP appears to share features with both migraine, in the recruitment of pain-processing regions by light, and FM, in the modulation of affective pain circuits by psychiatric symptom burden.

The differential activation of these cingulo-insular regions in response to light is biologically plausible given their established roles in pain and affective processing. The ACC has been implicated in the affective appraisal and emotional salience of pain(33, 34), whereas the aMCC has been associated with cognitive control, action selection, and behavioral responses to pain. (33, 34) The anterior insula contributes to interoceptive processing and the evaluation of behaviorally relevant sensory stimuli.(36, 37) Against this functional framework, COP + MHC+ participants showed greater light-evoked responses in the ACC and caudal aMCC, whereas participants without MHC showed greater responses in the rostral aMCC and anterior insula. The rostrocaudal aMCC findings warrant attention because prior light-evoked COP work treated the aMCC as a single region and did not report opposing subregional relationships with psychiatric symptoms.(19) Overall, cingulo-insular responses correlated with PTSD and depressive symptom severity in several regions, suggesting that affective symptom burden may influence neural responses to light within this network. Together, these exploratory findings demonstrate anatomically specific regional differences in light-evoked BOLD responses associated with screen-positive depressive and/or PTSD symptoms, although their functional significance cannot be determined from the present study and requires independent replication.

### Limitations.

As with any study, ours has several limitations. First, the sample was modest, groups were unequal, and participants were recruited from a single Veterans Affairs eye clinic, limiting precision and generalizability; 18 of 61 (30%) of scanned participants were also excluded, most commonly because of excessive fMRI motion artifact (n = 10). Second, MHC was defined using symptom-screening thresholds rather than diagnostic interviews, and the substantial overlap between depressive and PTSD symptoms precluded disorder-specific inference. Additionally, the absence of a psychiatric comparison group without COP limits the ability to determine whether the observed activation patterns reflect modulation of light-evoked processing in COP or broader neural correlates of psychiatric symptom burden. Third, because PHQ-9 and PCL-M correlations used parameter estimates extracted from clusters identified by the MHC group contrasts, these post hoc correlations were not statistically independent of cluster selection and should be interpreted descriptively. Finally, the cross-sectional design precludes causal inference, and potential confounding by medication use, co-occurring pain conditions including migraine, TBI, and other clinical factors could not be comprehensively adjusted for in this sample. Larger independent cohorts are needed to replicate these regional findings.

## Conclusion

Among individuals with COP, screen-positive depressive and/or PTSD symptoms were associated with bidirectional regional differences in light-evoked BOLD responses within the right ACC, aMCC, and anterior insula. The opposing rostrocaudal aMCC findings and region-specific symptom associations suggest that psychiatric symptom burden may contribute to heterogeneity in light-evoked brain responses rather than a uniform increase in activation across affective pain-processing regions. These exploratory findings require replication in larger cohorts using independently defined regions and appropriate correction for multiple comparisons. Clinically, these findings support considering psychiatric symptoms alongside ocular and pain-related features in clinical assessment. A better understanding of how these factors shape the neural mechanisms underlying COP may ultimately help identify biologically distinct patient subgroups and guide more individualized treatment strategies.

## Supplementary Files

This is a list of supplementary files associated with this preprint. Click to download.

- SupplementaryTable1.docx

## Figures and Tables

**Figure 1:**
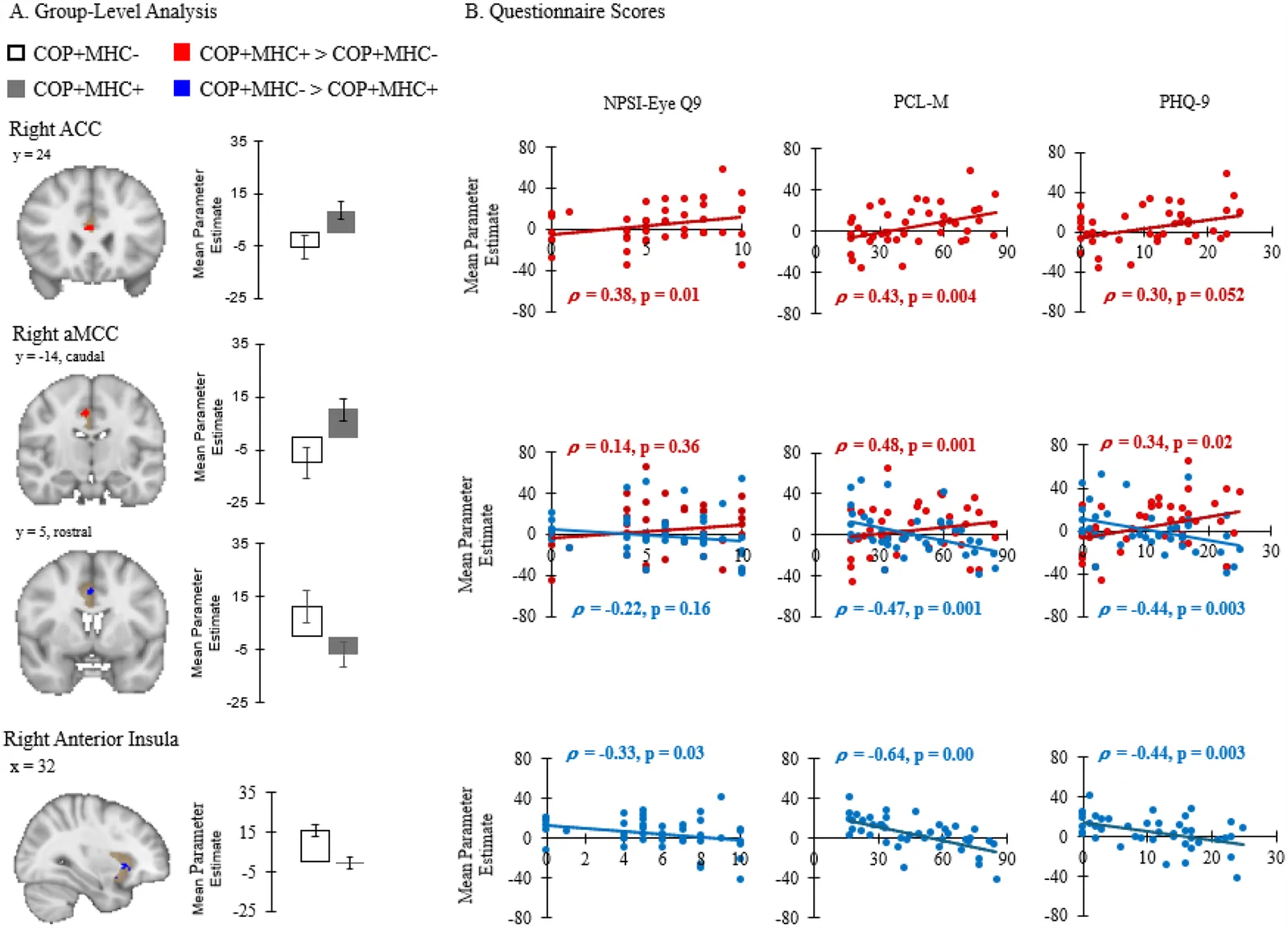
Light-evoked BOLD responses within predefined affective pain-processing regions of interest (ROIs) in participants with chronic ocular pain (COP) according to mental health comorbidity (MHC). (A) Exploratory ROI group-level Z-statistic maps for the contrasts COP+MHC+ > COP+MHC− (red) and COP+MHC− > COP+MHC+ (blue) show clusters exceeding the exploratory voxelwise threshold within the right ACC, right aMCC, and right anterior insula (voxelwise threshold Z>2.3, uncorrected). ROI masks were generated in MNI152 standard space using Harvard-Oxford probability maps thresholded at ≥20%. Hemisphere-specific masks were generated by dividing atlas-derived ROI masks at the midsagittal plane. The two spatially distinct right aMCC clusters are labeled rostral and caudal according to their relative anterior-posterior anatomical locations. Adjacent bar plots show mean contrast of parameter estimates (COPEs) ± SEM for each group. (B) Scatterplots depict correlations between subject-level parameter estimates (PEs) extracted from each retained cluster and clinical measures (NPSI-Eye Q9, PCL-M, and PHQ-9). Colored lines illustrate within-group least-squares fits (red = COP+MHC+, blue = COP+MHC−); Spearman correlation coefficients (*ρ*) and associated p values for the full cohort are reported in the text.

**Table 1:** Population demographics and comorbidities grouped by presence or absence of mental health comorbidities (MHC).

Characteristic	Full Cohort (N = 43)	COP + MHC+ (N = 30)	COP + MHC-(N = 13)	p-value
Demographics				
Age, years (mean ± SD)	55.9 ± 11.5	56.7 ± 11.5	54.2 ± 11.9	0.53
Sex, female	16 (37%)	9 (30%)	7 (54%)	0.18
Race				
White	32 (74%)	23 (77%)	9 (69%)	0.31
Black	10 (23%)	7 (23%)	3 (23%)	
Ethnicity, Hispanic	23 (53%)	15 (50%)	8 (62%)	0.53
Comorbidities				
Rheumatoid arthritis	4 (9%)	3 (10%)	1 (8%)	1.00
Non-rheumatoid arthritis	5 (12%)	4 (13%)	1 (8%)	1.00
Sleep apnea	22 (51%)	16 (53%)	6 (46%)	0.75
CPAP use	20 (47%)	16 (53%)	4 (31%)	0.20
BPH	7 (16%)	6 (20%)	1 (8%)	0.41
Rosacea	5 (12%)	3 (10%)	2 (15%)	0.63
TBI	10 (23%)	9 (30%)	1 (8%)	0.24
Migraine	15 (35%)	12 (40%)	3 (23%)	0.49
Hypertension	15 (35%)	12 (40%)	3 (23%)	0.49
Hypercholesterolemia	16 (37%)	11 (37%)	5 (38%)	1.00
Medication use				
NSAIDs	14 (33%)	9 (30%)	5 (38%)	0.73
Fish oil	13 (30%)	10 (33%)	3 (23%)	0.72
Multivitamins	22 (51%)	15 (50%)	7 (54%)	1.00
Antidepressant	18 (42%)	15 (50%)	3 (23%)	0.18
Anxiolytic	19 (44%)	15 (50%)	4 (31%)	0.32
Analgesic	25 (58%)	18 (60%)	7 (54%)	0.75
Antihistamine	19 (44%)	15 (50%)	4 (31%)	0.32
Oral Neuromodulators (Gabapentin/Pregabalin/Duloxetine)	11 (26%)	7 (23%)	4 (31%)	0.71
Health Score				
Good	29 (67%)	19 (63%)	10 (77%)	0.26
Fair	12 (28%)	10 (33%)	2 (15%)	
Smoking status				
Never	21 (49%)	14 (47%)	7 (54%)	0.72
Past	20 (47%)	15 (50%)	5 (39%)	0.56
Current	2 (5%)	1 (3%)	1 (8%)	0.82

COP = chronic ocular pain; MHC = mental health comorbidity; SD = standard deviation; CPAP = continuous positive airway pressure; BPH = benign prostate hyperplasia; TBI = traumatic brain injury; NSAIDs = nonsteroidal anti-inflammatory drugs.

**Table 2:** Clinical symptom measures and clinical exam findings in participants with chronic ocular pain (COP), grouped by presence or absence of mental health comorbidities (MHC).

Measure	Full Cohort (N = 43)	COP + MHC+ (N = 30)	COP + MHC− (N = 13)	p-value
Ocular pain-related				
Current eye pain level	4.1 ± 2.6	4.4 ± 2.6	3.5 ± 2.6	0.28
Worst eye pain, 1-week recall	6.4 ± 2.7	6.8 ± 2.4	5.4 ± 3.0	0.10
Average eye pain, 1-week recall	4.9 ± 2.6	5.3 ± 2.5	3.8 ± 2.6	0.08
NPSI-Eye total	31.5 ± 19.4	34.2 ± 18.9	25.4 ± 19.9	0.18
Non-ocular pain-related				
Current pain level	4.3 ± 2.8	4.7 ± 2.5	3.3 ± 3.3	0.13
Worst pain, 1-week recall	5.9 ± 3.2	6.8 ± 2.6	3.9 ± 3.8	**0.02**
Average pain, 1-week recall	4.9 ± 2.9	5.6 ± 2.5	3.4 ± 3.3	**0.047**
Photoallodynia-related				
NPSI-Eye Q9 (light sensitivity)	5.7 ± 3.1	6.1 ± 2.8	4.6 ± 3.6	0.15
VLSQ-8	27.5 ± 7.3	28.3 ± 7.0	25.6 ± 8.1	0.28
OSDI Q1 (light sensitivity)	2.8 ± 1.2	2.9 ± 1.1	2.6 ± 1.5	0.55
Dry eye disease-related				
OSDI	54.7 ± 20.8	60.6 ± 19.5	41.0 ± 17.4	**0.003**
DEQ-5	14.2 ± 3.4	14.6 ± 3.4	13.5 ± 3.6	0.35
Mental health-related				
PHQ-9	11.0 ± 8.1	15.2 ± 5.8	1.2 ± 1.4	**< 0.001**
PCL-M	46.3 ± 21.9	56.9 ± 17.3	22.0 ± 5.7	**< 0.001**
Ocular surface signs				
Anterior blepharitis	1.1 ± 0.9	1.2 ± 1.0	0.8 ± 0.7	0.31
Eyelid vascularity	0.9 ± 0.8	1.0 ± 0.8	0.7 ± 0.9	0.32
Palpebral hyperemia	0.7 ± 0.6	0.7 ± 0.5	0.7 ± 0.6	0.97
Conjunctival papillae	0.2 ± 0.5	0.2 ± 0.5	0.2 ± 0.4	0.76
Meibomian gland plugging	1.6 ± 0.9	1.6 ± 0.9	1.5 ± 1.0	0.76
Meibum quality	1.4 ± 1.6	1.6 ± 1.6	1.0 ± 1.5	0.23
TBUT (s)	6.8 ± 3.7	7.2 ± 3.8	5.7 ± 3.5	0.22
Corneal staining	2.8 ± 3.6	2.6 ± 2.7	3.2 ± 5.2	0.72
Schirmer's (mm)	10.9 ± 8.0	8.9 ± 5.3	9.3 ± 5.1	0.30
Anesthetic challenge (0–10 NRS)*				
Baseline	3.2 ± 2.9	3.8 ± 2.8	1.8 ± 2.7	**0.03**
Post-anesthetic	2.1 ± 2.4	2.7 ± 2.7	0.8 ± 1.0	**0.02**
Post-anesthetic minus baseline	−1.1 ± 2.3	−1.1 ± 2.5	−1.0 ± 1.8	0.86
% with any pain post-anesthetic	26 (60.5%)	20 (66.7%)	6 (46.2%)	0.31
Pain ratings during fMRI (0–100 NRS)*				
Eye pain, *ON* state	29.1 ± 35.1	34.4 ± 36.7	16.8 ± 28.4	0.13
Eye unpleasantness, *ON* state	43.3 ± 35.3	48.2 ± 36.0	31.9 ± 31.9	0.17

Values from the more abnormal eye (higher scores for inflammatory and staining measures; lower scores for TBUT and Schirmer’s) were selected for analysis. Bolded values indicate significance at p < 0.05. Ocular pain intensity (0–10 NRS scale) was recorded before and after instillation of 1 drop of 0.5% proparacaine in each eye. Scores were analyzed at baseline, post-anesthetic application, and as the difference (post-anesthetic minus baseline).

* Clinical pain ratings were collected using a 0–10 numerical rating scale (NRS), whereas in-scanner light-evoked pain and unpleasantness were rated on a 0–100 NRS to provide greater quantitative granularity for fMRI analyses.

Abbreviations: COP = chronic ocular pain; MHC = mental health comorbidity; NPSI-Eye = Neuropathic Pain Symptom Inventory–Eye; NRS = numerical rating scale; Q = question; VLSQ-8 = Visual Light Sensitivity Questionnaire–8; OSDI = Ocular Surface Disease Index; DEQ-5 = 5-Item Dry Eye Questionnaire; PHQ-9 = Patient Health Questionnaire-9; PCL-M = PTSD Checklist-Military Version; s = second; TBUT = tear breakup time.

**Table 3:** Group-level Differences in Light-evoked BOLD Activation Between Participants With and Without Comorbid Mental Health Conditions.

		COP + MHC + > COP + MHC−						COP + MHC− > COP + MHC+				
Brain region		Side	Z-max	PeakMNI (mm)			Cluster extent (mm^3^)	Z-max	Peak MNI (mm)			Cluster extent (mm^3^)
				X	Y	Z			X	Y	Z	
Anterior Cingulate C		R	11.36	4	24	16	152					
Anterior Mid-Cingulate C	Caudal	R	16.87	7	−14	43	144					
	Rostral							10.79	2	5	40	224
Anterior Insular C		R						11.97	32	24	−6	200
Amygdala		B										

Brain regions containing clusters exceeding the exploratory voxelwise threshold for between-group differences in light-evoked blood oxygen level–dependent (BOLD) activation in participants with chronic ocular pain and comorbid mental health conditions (COP + MHC+) compared with those without such comorbidities (COP + MHC−). For each contrast, analyses were restricted to voxels showing positive light-evoked activation exceeding the specified voxelwise threshold in the reference group (COP + MHC+ for the first contrast, COP + MHC− for the second), allowing identification of regions where activation was reduced relative to the reference group.

Contrast of parameter estimates (COPEs), indexing the magnitude and direction of between-group differences in BOLD signal, were computed voxelwise and averaged within clusters for visualization. Statistical maps were evaluated using an exploratory voxelwise threshold of Z > 2.3 with a minimum cluster extent of six contiguous voxels; no correction for multiple comparisons across voxels or ROIs was applied. For each region, the peak Z-statistic (Z-max), corresponding peak MNI coordinates (x, y, z), and suprathreshold cluster extent (mm^3^) are provided.

## Data Availability

The data supporting the findings of this study are available from the corresponding author upon reasonable request, subject to applicable institutional approvals and protections for participant confidentiality.
